# EphA5 and EphA6: regulation of neuronal and spine morphology

**DOI:** 10.1186/s13578-016-0115-5

**Published:** 2016-08-02

**Authors:** Gitanjali Das, Qili Yu, Ryan Hui, Kenneth Reuhl, Nicholas W. Gale, Renping Zhou

**Affiliations:** 1 Susan L. Cullman Laboratory for Cancer Research, Ernest Mario School of Pharmacy, Rutgers University, Piscataway, NJ 08854 USA; 2 Department of Pharmacology and Toxicology, Ernest Mario School of Pharmacy, Rutgers University, Piscataway, NJ 08854 USA; 3 Regeneron Pharmaceuticals, Inc., Tarrytown, NY 10591 USA

**Keywords:** EphA5, EphA6, Golgi staining, Cortex, Dendrite, Spine

## Abstract

**Background:**

The Eph family of receptor tyrosine kinases plays important roles in neural development. Previous studies have implicated Eph receptors and their ligands, the ephrins, in neuronal migration, axon bundling and guidance to specific targets, dendritic spine formation and neural plasticity. However, specific contributions of EphA5 and EphA6 receptors to the regulation of neuronal cell morphology have not been well studied.

**Results:**

Here we show that deletion of EphA5 and EphA6 results in abnormal Golgi staining patterns of cells in the brain, and abnormal spine morphology.

**Conclusion:**

These observations suggest novel functions of these Eph receptors in the regulation of neuronal and spine structure in brain development and function.

## Background

The Eph family is the largest group of related receptor tyrosine kinases known, consisting of 16 members in the vertebrate genome [1]. These receptors, interacting with their ligands, the ephrins, regulate many different functions during embryonic development and in postnatal life, including tissue segmentation, angiogenesis, axonal guidance and synaptic plasticity [2–8]. The Eph receptors are divided into two groups (10 EphAs and 6 EphBs) according to their ligand binding preferences; most EphAs bind to the GPI-linked ephrin-A ligands while EphBs bind to the transmembrane ephrin-B ligands. However, there is some promiscuity in their binding affinities, viz., EphA4 bind to ephrin-B ligands and EphB2 shows attraction towards ephrin-A5 ligand [9–11].

The cerebral cortex regulates highly developed behavioral and cognitive functions [12–14]. Thus, its precise development is essential for a functional brain. The Ephs/ephrins family of molecules plays an important role in the development of the cortex as most of these molecules are highly expressed in this brain region. Studies have shown that cortical compartmentalization closely follows the expression profile of various Eph/ephrin during development [15–18]. The molecular gradients of different Ephs/ephrins function in a bidirectional manner wherein their varying dosage determines the levels of either attractive or repulsive force experienced by the developing neuron during migration, synapse formation and function [19–22]. EphA5 and EphA6 are highly expressed in different layers of the cortex [23–25]. We have shown previously that interfering with EphA5 function using transgenic expression of the truncated receptor lacking the intracellular domain resulted in deficits in spatial navigation and impairment in active avoidance, coupled with a decrease in striatal dopamine and serotonin concentrations [26]. We also showed that mice with EphA5 deletion had reduced level of intermale aggression, similar to that of the deletion of one of the major ligands ephrin-A5 [27, 28]. Genetic inactivation of EphA6 also led to an impairment in learning and memory [29]. Thus it is clear that EphA5 and EphA6 receptors play key roles in brain development and/or behavior regulation. However, specific neuronal changes induced by the deletion of EphA5 or EphA6 have not been well documented. In the present study, we examined neuronal morphology in the brains of mice with genetic inactivation of EphA5 and EphA6 receptors. We report here that in both knockout strains, Golgi staining revealed large neuronal aggregates that were not present in the wild type brains. In addition, dendritic spine morphology of the cerebral cortical neurons was severely altered in these mice. These observations reveal significant deficiencies in neuronal morphology, consistent with functional deficits observed in behavioral studies of these mice.

## Methods

### Mouse strains

#### EphA5 LacZ/LacZ mice

Generation of the EphA5 mice has been reported earlier [25, 30].

#### EphA6 LacZ/LacZ mice

Briefly, EphA6 deletion mouse strain was generated as the following: the EphA6 receptor genomic DNA isolated from a 129SV mouse genomic library screen was cloned into the TM-Zen_UB1 Vector cassette. The LacZ coding sequence was fused in frame to exon 3 of EphA6 at the endogenous Bam H1 site to produce a targeting vector containing a human Ubiquitin C promoter-driven neomycin-resistant gene that was used to target ES cells which were implanted into female mice. Mice generated were screened for the wild type, knockout and heterozygous allele within the colony using the following primers for PCR. Wild type primers (5′ATCCCCAAAGAGTAGGTTCC3′; 5′CCTCACGGATTTCAGTGTTGAG3′) generated a PCR product of a molecular weight of 455 bp, while the knockout primers (5′ ATCCCCAAAGAGTAGGTTCC3′; 5′GTCTGTCCTAGCTTCCTCACTG3′) produced a 449 base pair product.

#### Double knockout mice

The EphA5 and the EphA6 knockout mice were interbred to produce double knockout (KO) mice. These mice have no obvious physical and reproductive abnormality.

Mice were housed under standard conditions as outlined in the Guidelines for the Care and Use of Laboratory Animals of Rutgers University.

### β-galactosidase staining

Expression of the knocked-in β-galactosidase gene was observed following the protocol described previously [25]. Briefly, 60 day-old mice were anesthetized using ketamine/xylazine as approved under the institutional guideline. Brain was dissected out quickly, frozen in OCT on dry-ice and stored in −80 °C until sectioning. 10 μm sections were mounted on superfrost plus slides, lightly fixed for a minute in 2 % paraformaldehyde/0.5 % glutaraldehye solution in PBS followed by brief washes in PBS three times and allowed to develop for 18 h in a reaction buffer containing 1 mg/ml X-Gal, 5 mM Potassium Ferricyanide, 5 mM Potassium Ferrocyanide, 2 mM Magnesium Chloride, 0.01 % Sodium Deoxycholate and 0.02 % NP-40 in a 37 °C incubator. After color development, sections were dehydrated, coverslipped in permount and dried under a hood overnight before imaging under microscope.

### Golgi staining

Two different protocols of Golgi staining were done for the present study as already published [31]. For the first experiment, mice were perfused with 4 % paraformaldehyde (PFA), pH 7.4. The brain was dissected out, cut in half at the junction between the cortex and midbrain and further incubated in the PFA solution for a further 10 min, followed by immersion in the Golgi solution (FD Neurotechnologies, Rapid Golgi Kit). The Golgi solution was changed after 6 h, and the brain was kept immersed as such for two weeks before development according to the instructions by the manufacturer. For the second set of experiment, fresh brain without PFA perfusion was immersed in the Golgi solution for one week. The brains were sectioned at 250 μm thickness in a vibrating microtome and color developed following the instructions from the manufacturer and imaged under microscope after drying.

### Microscopy

Bright field images were obtained using a Zeiss Axiovert 200 M microscope using the ProRes software for the spine pictures and with the Openlab software for the dendrites and dendrites were drawn using the Neurolucida software. The different color of the dendrites indicate different starting points while drawing in the Neurolucida and thus differentiate primary and secondary dendrites.

## Results

In an effort to examine roles of EphA5 and EphA6 in cerebral cortical development, we examined the expression of these two receptors and the effects of inactivation on neuronal structure using Golgi staining and immunohistochemistry.

### EphA5 and EphA6 expression in the adult brain

**Fig. 1:**
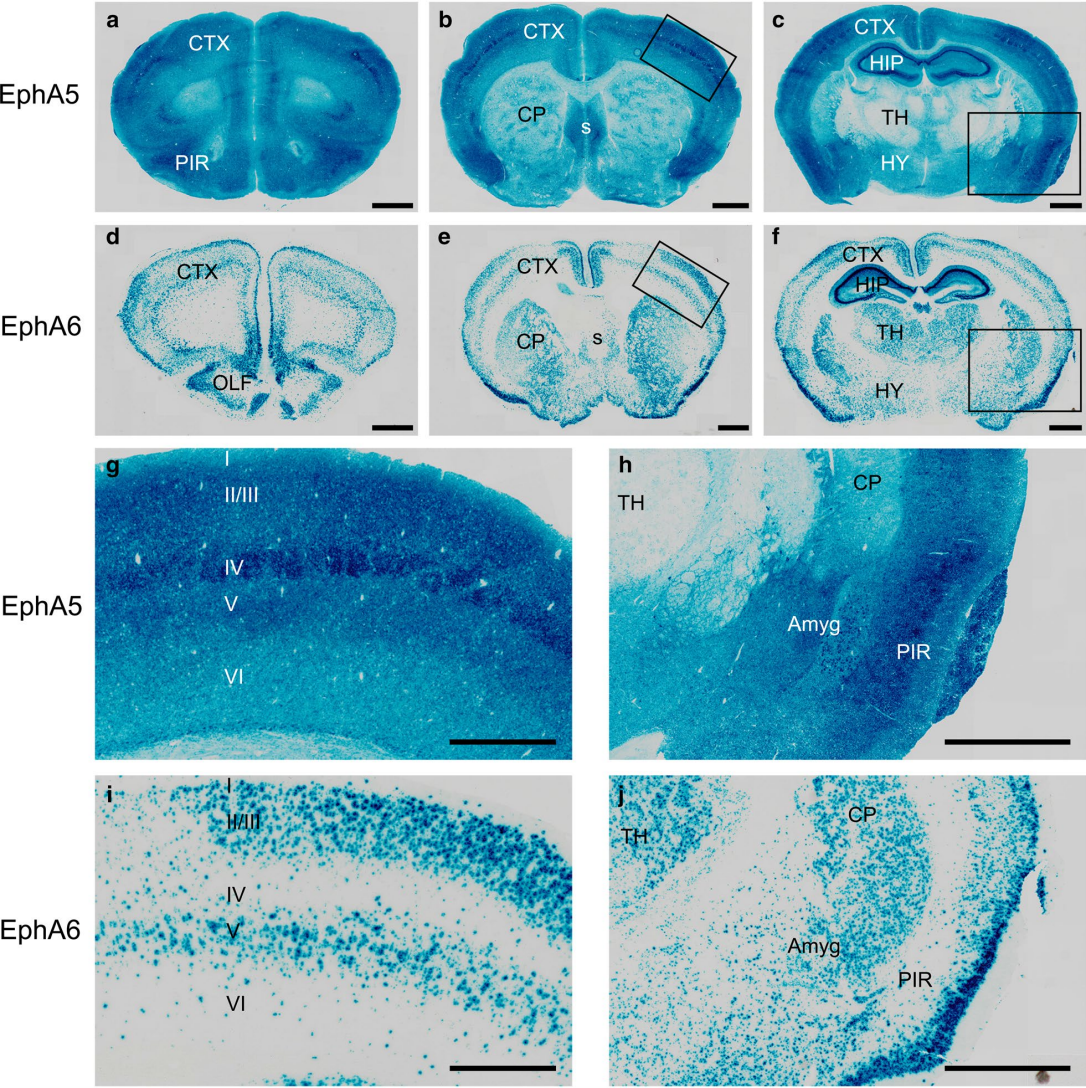
Representative images of the beta-galactosidase staining in different regions of the mouse brain showing the expression of EphA5 (**a**–**c**, **g**, **h**) and EphA6 (**d**–**f**, **i**, **j**) genes. Highest expression of both genes is present in the cortical regions with also diffuse presence in the thalamus, hypothalamus, hippocampus and amygdala as well (n = 3 for both EphA5 and EphA6 brains). *Amyg* amygdala; *CP* Caudate Putamen; *CTX* cerebral cortex; *HIP* hippocampus; *HY* hypothalamus; *TH* thalamus; *s* septum; *OLF* olfactory area; *PIR* pyriform cortex; *Scale bars* **a**–**f**, 1 mm; **g**, **i**, 0.5 mm: **h**, **j**, 1 mm

To compare expression of EphA5 and EphA6 in the adult brain, sections of the heterozygous and homozygous adult EphA5 and EphA6-LacZ gene replacement mice were examined for β-galactosidase expression using LacZ staining [25, 32]. A detailed study of EphA5 expression from embryonic day (e) 9 to adult has been performed previously in our laboratory [25] and the expression in the 2 months-old brains were reexamined in parallel with EphA6 (see the following paragraph) for comparison in this study. These analyses revealed significant levels of EphA5 expression in the cerebral cortex, amygdala, piriform cortex, and hippocampus (Fig. 1). Cerebral cortex showed a diffused expression throughout with more distinguished signals in the cortex layers II/III, IV, and V (Fig. 1g). Very little expression was visible in the septum, hypothalamus and cerebellum with faint expression in the thalamus and striatum (Fig. 1). This general pattern of expression was maintained from birth to adult (Fig. 1 and [25]).

Since the morphological abnormality was not apparent until 1 month of age in the EphA6 knockout mice (see the following section and data not shown), we examined EphA6 expression in the brains from the age of postnatal day 1, 10 and 2 months old mice, high EphA6 expression was shown by LacZ staining in most regions of the brain, viz., orbital cortex, olfactory lobes with stripes in the internal plexiform layer, lateral olfactory tubercle, septum, striatum, thalamus, hypothalamus and cerebellum. In the cerebral cortex it is most highly expressed in layers II, III and V (Fig. 1), with considerable overlap with that of EphA5. This pattern of expression was similar in the brains of P0, P10 and 2 months-old mice (Fig. 1 and data not shown).

### Morphological analyses of cortical neurons of the EphA5^−/−^, EphA6^−/−^ and the double EphA5^−/−^EphA6^−/−^ mice

**Fig. 2:**
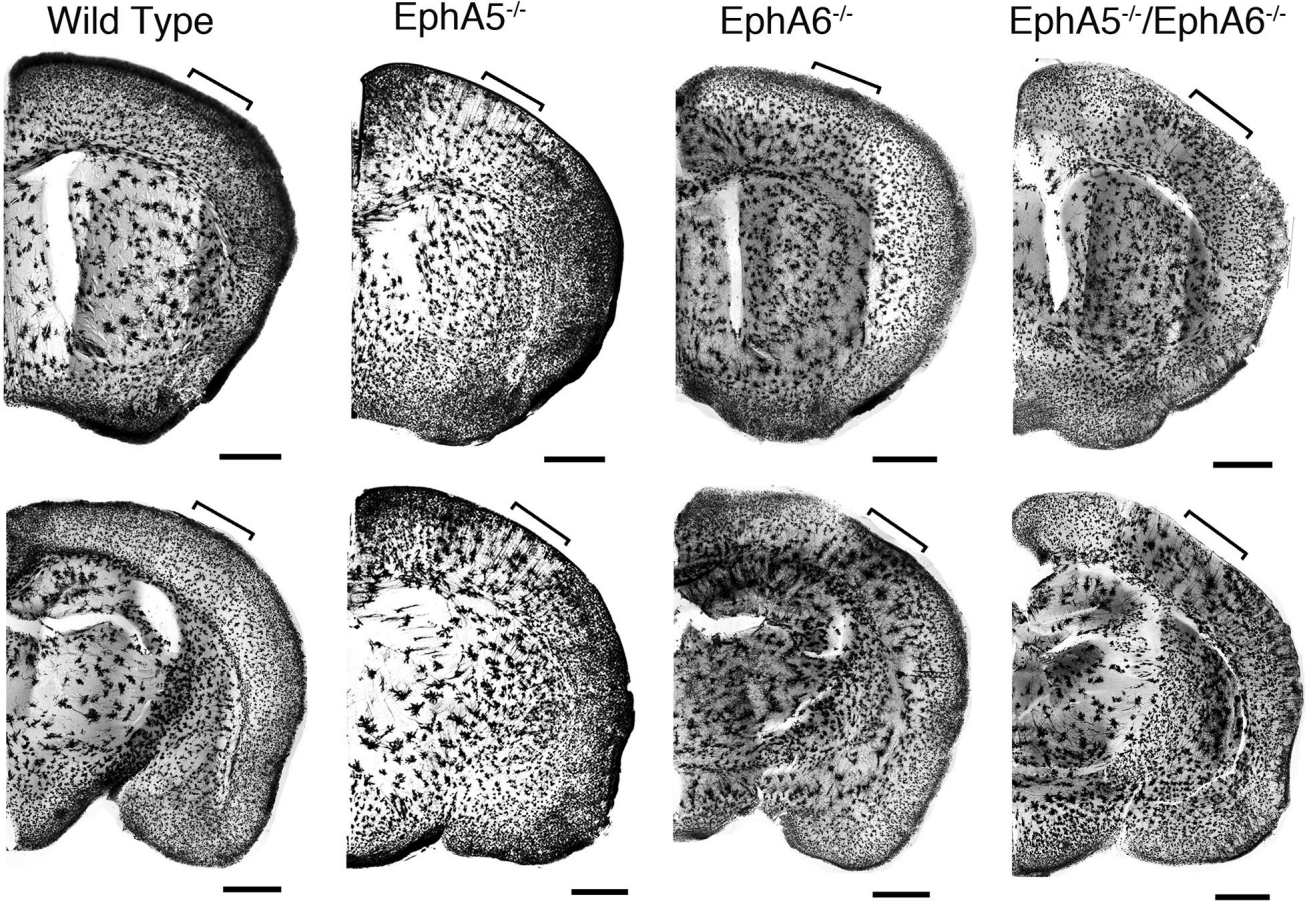
Representative images of Golgi staining done in paraformaldehyde perfused brains showing aggregation of neurons in the frontal cortical (*upper panels*) and mid-cortical (*lower panels*) regions of both EphA5 and EphA6 KO brains. The double knockout (DKO) of EphA5 and EphA6 did not show a more pronounced effect on this aggregation phenomenon. The *bracket areas* show approximate locations of the cortex that are examined in higher magnification in Fig. 3. *Scale bars* 1 mm

**Fig. 3:**
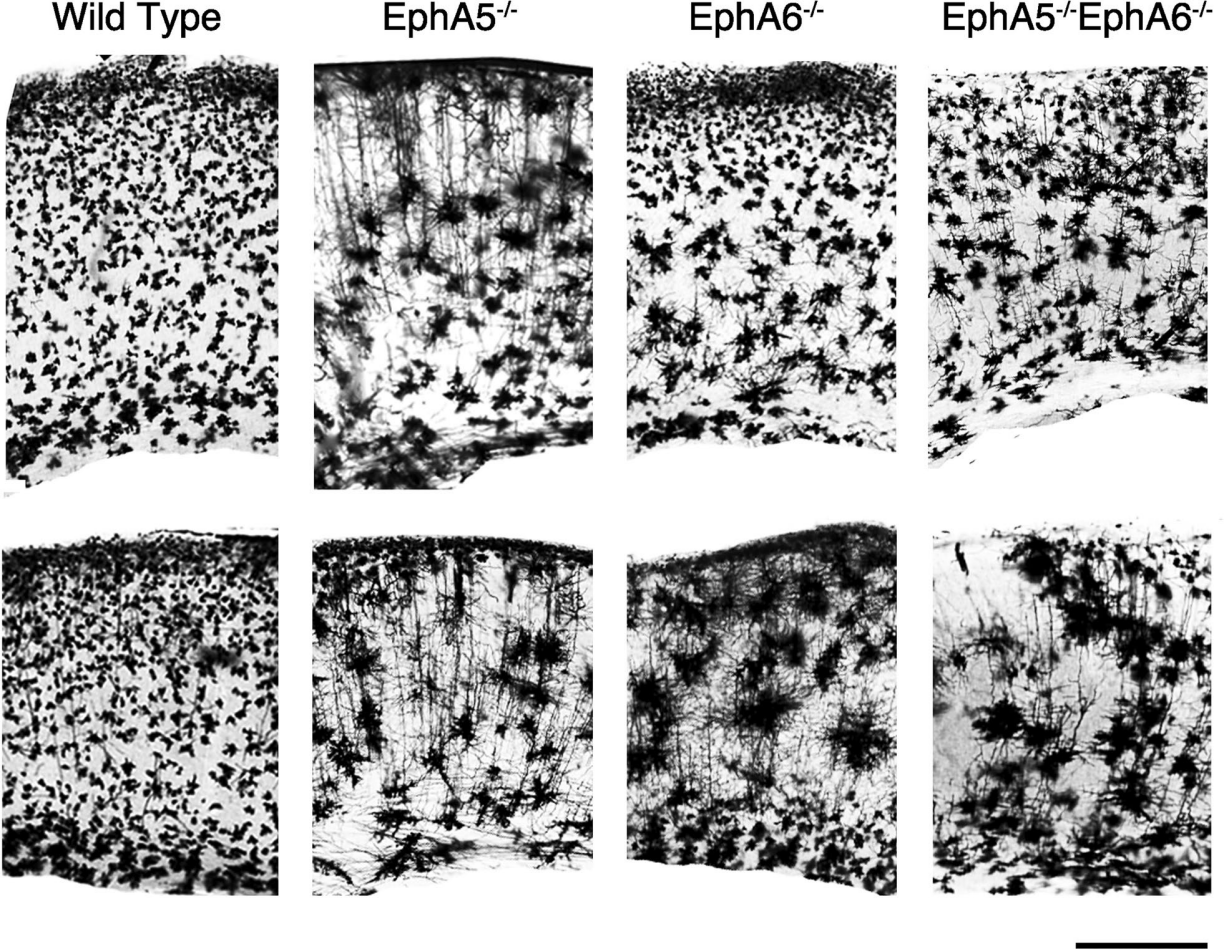
Representative higher magnification images of Golgi staining done in paraformaldehyde perfused brain showing aggregation of neurons at the frontal cortical (*upper panels*) and the mid-cortical (*lower panels*) regions of both EphA5 and EphA6 KO brains. The double knockout (DKO) of EphA5 and EphA6 did not show a more pronounced effect on this aggregation phenomenon. *Scale bar* 500 µm

**Fig. 4:**
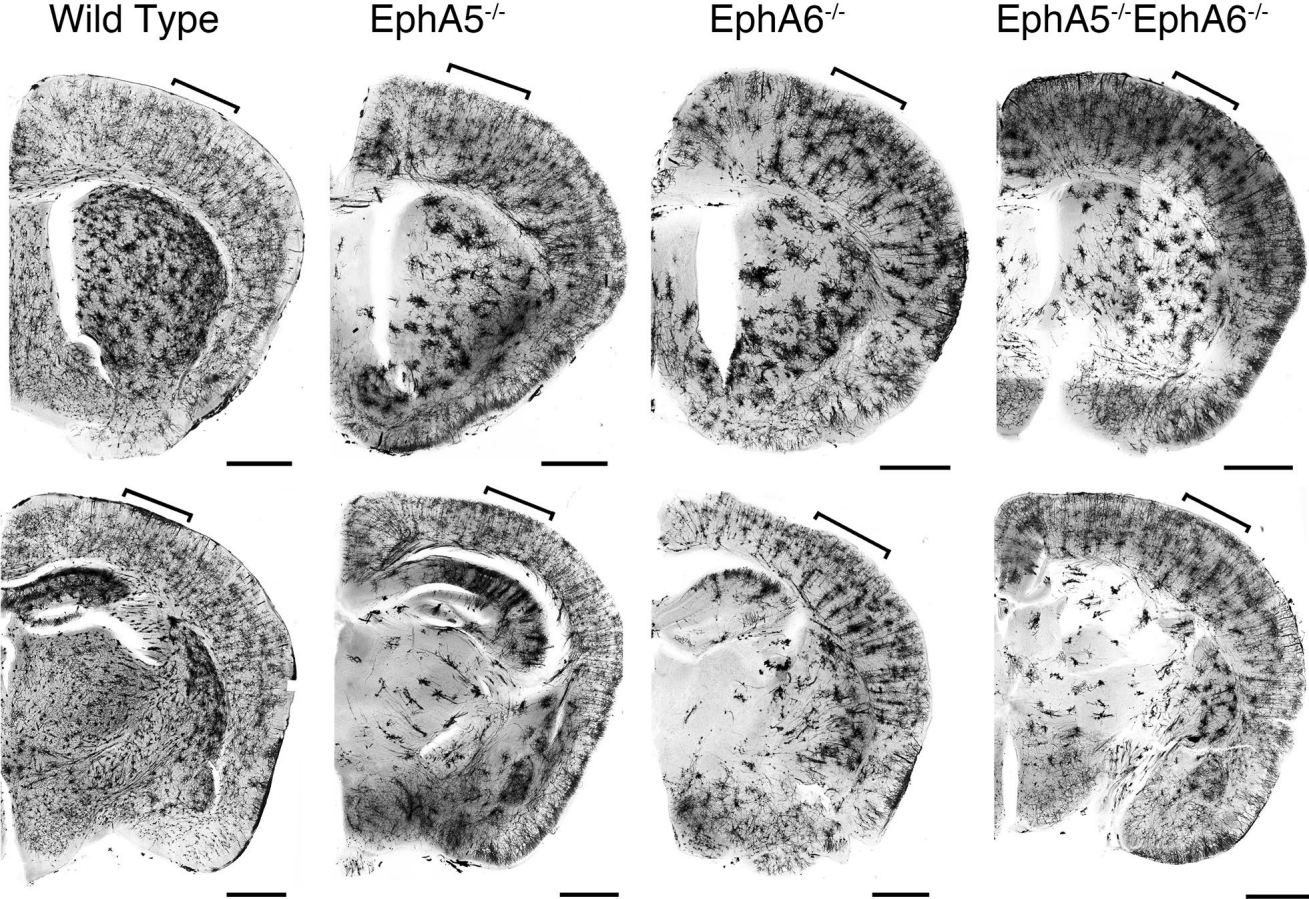
Representative images of Golgi staining done in fresh non-paraformaldehyde perfused brains showing aggregation of neurons in the frontal cortical (*upper panels*) and the mid-cortical (*lower panels*) regions of both EphA5 and EphA6 KO brains. The double knockout (DKO) of EphA5 and EphA6 did not show a more pronounced effect on this aggregation phenomenon. Golgi staining of the fresh brains clearly showed that the aggregation phenomenon is mostly a neuronal effect. The *bracket areas* show approximate locations of the cortex that are examined in higher magnification in Fig. 5. *Scale bars* 1 mm

**Fig. 5:**
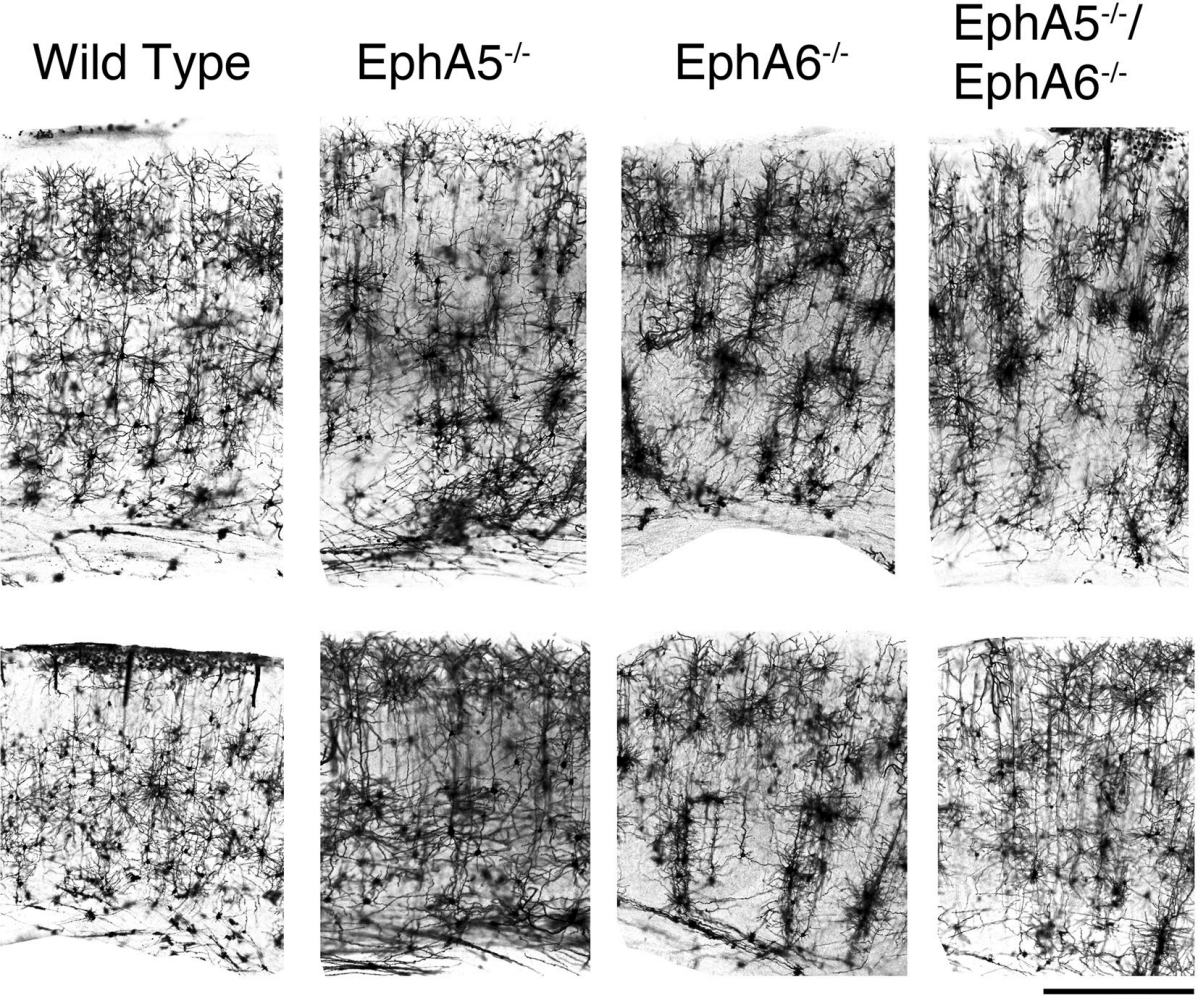
Representative higher magnification images of Golgi staining done in fresh non- paraformaldehyde perfused brains showing aggregation of neurons at the frontal cortical (*upper panels*) and the mid-cortical (*lower panels*) regions of both EphA5 and EphA6 KO brains. The double knockout (DKO) of EphA5 and EphA6 did not show a more pronounced effect on this aggregation phenomenon. Golgi staining of the fresh brains clearly showed that the aggregation phenomenon is mostly a neuronal effect. *Scale bar* 500 µm

To examine effects of EphA5 and EphA6 deletion on neuronal morphology, we performed Golgi staining on the brains of EphA5 and EphA6 knockout mice. We have initially analyzed EphA6 KO brains at the ages of 1 week, 1, 2 and 5–6 months. At 1 week, Golgi staining of the paraformaldehyde perfused EphA6^−/−^ brains showed no striking difference from the wild type (data not shown). Somewhat increased size of cell aggregates was observed in the brains of 1 month-old EphA6-null animal. However, by 2 months, strikingly large abnormal aggregates of cells were detected in parts of the cerebral cortex of the EphA6^−/−^ animals (Fig. 2). This phenotype is maintained in 5–6 months old EphA6^−/−^ brains. Thus more detailed analyses were performed primarily using brains of various strains of 2 months old animals. Our analyses revealed that significantly larger cell aggregates were present in the cerebral cortex of the 2 months-old EphA5^−/−^, EphA6^−/−^ and EphA5^−/−^EphA6^−/−^ mouse brains, compared with that of the wild type controls (Figs. 2, 3). The wild type mouse brains showed relatively even distribution of cells in the cerebral cortex. In contrast, the EphA5^−/−^ mouse brains exhibited a clumping phenotype of the cells in the cerebral cortex (Figs. 2, 3). This effect was more pronounced in the EphA6^−/−^ mouse brain. Clumping was seen in many cortical areas, but most prominently in the sensory cortex (Bracketed area in Figs. 2, 3, 4, 5). The double knockout mouse brain showed a similar morphological phenotype to the EphA6^−/−^ mice.

The protocol using paraformaldehyde perfusion results in staining of many glial cells as well as neurons but with few of their dendrites [31]. To specifically examine the cytoarchitecture of neurons in the knockouts, we performed Golgi staining of brains in all genotypes without prefixation (Figs. 4, 5). With this protocol, extensive dendrites as well as neuronal cell somata are readily detectable. A similar aggregation phenotype was observed as in the pre-fixed brains, although the aggregates appear to be somewhat smaller, suggesting that glial cells, which are not stained well in this second protocol, also contribute to the aggregation. The Golgi brain sections without prefixation showed that cortical neurons of large aggregates can be observed in both the EphA5^−/−^ and the EphA6^−/−^ brains, with a more drastic effect observed in the EphA6^−/−^ brains. In the EphA5^−/−^ mouse brain, neuronal aggregates were observed most prominently in the deeper cortical layers but also in upper layers as well, where significant EphA5 expression is revealed by the LacZ staining (Fig. 1). The EphA6^−/−^ brain showed a more marked phenotype with large aggregates in brain regions which coincide with high EphA6 expression (Fig. 1). The double knockout mouse brain showed a very similar phenotype as the EphA6^−/−^ mice, suggesting that EphA6 plays a dominant role in regulating cellular morphology.

### The cell aggregates include both neurons and NeuN-negative cells

**Fig. 6:**
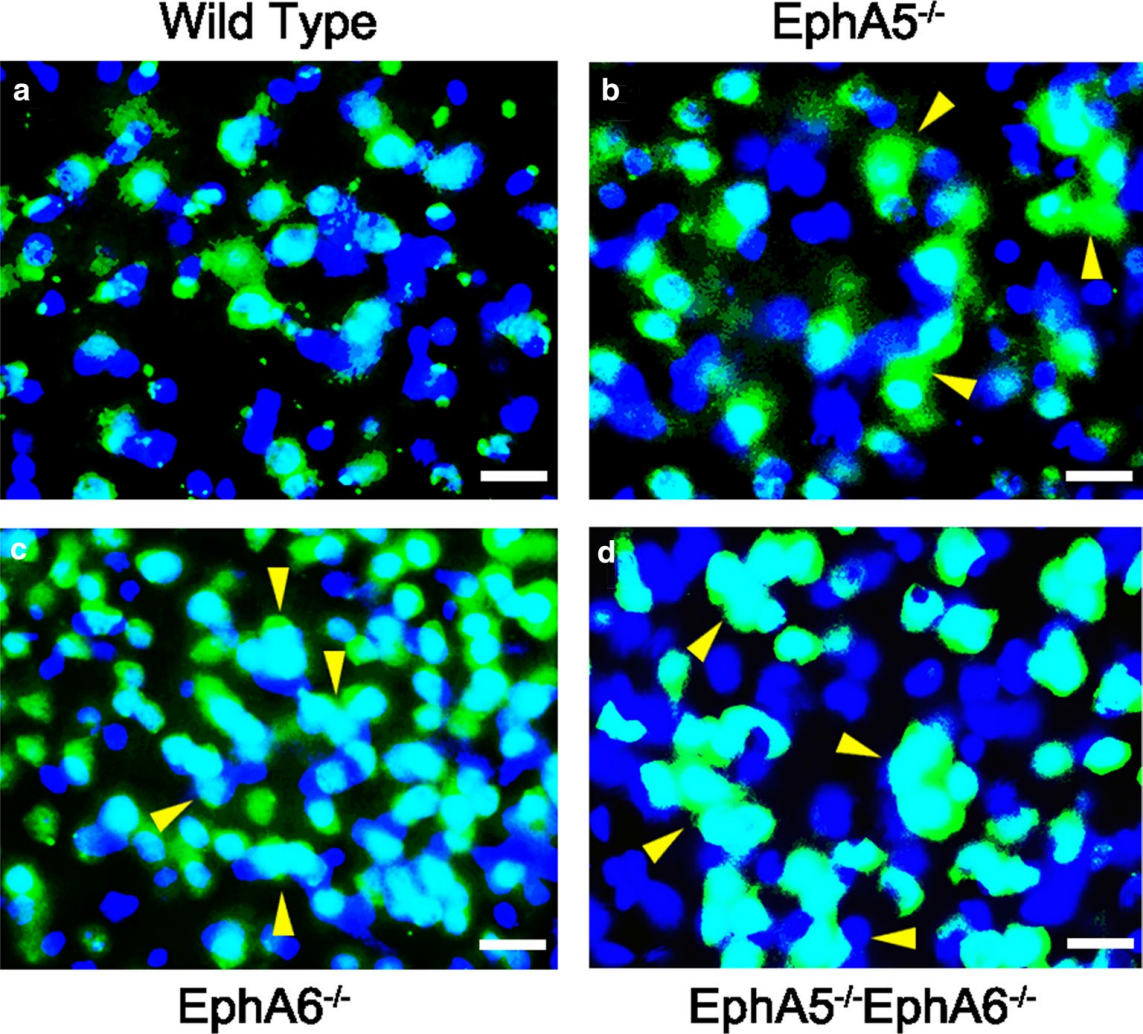
Double immunostaining showed the presence of neuronal clumps (NeuN in *green*) in the cortical layer 5 in all the knockout types. Most of the clumps were neuronal in origin with a few NeuN-negative cells as well (*yellow arrows*). **a** wild type; **b** EphA5^−/−^; **c** EphA6^−/−^; **d** EphA5^−/−^EphA6^−/−^. *Scale bars* 20 µm

To determine whether the cell aggregates include both neurons and non-neuronal cells, we performed immunostaining of the brain sections with the neuronal marker NeuN to identify neurons combined with DAPI to detect all cells in the cortex. These experiments showed the cell aggregates revealed by Golgi staining contained both NeuN-positive and NeuN-negative nuclei in the knockouts cortex (Fig. 6), suggesting that both neurons and possibly non-neuronal cells are involved in the abnormal cell aggregates. In addition, the wild type cortical nuclei were more evenly spread out than the knockouts, these data corroborate the Golgi data as shown in Figs. 2, 3, 4, 5.

### Basal dendrite morphology of cortical layer 5 neurons of the EphA5^−/−^, EphA6^−/−^ and the EphA5^−/−^EphA6^−/−^ mice

**Fig. 7:**
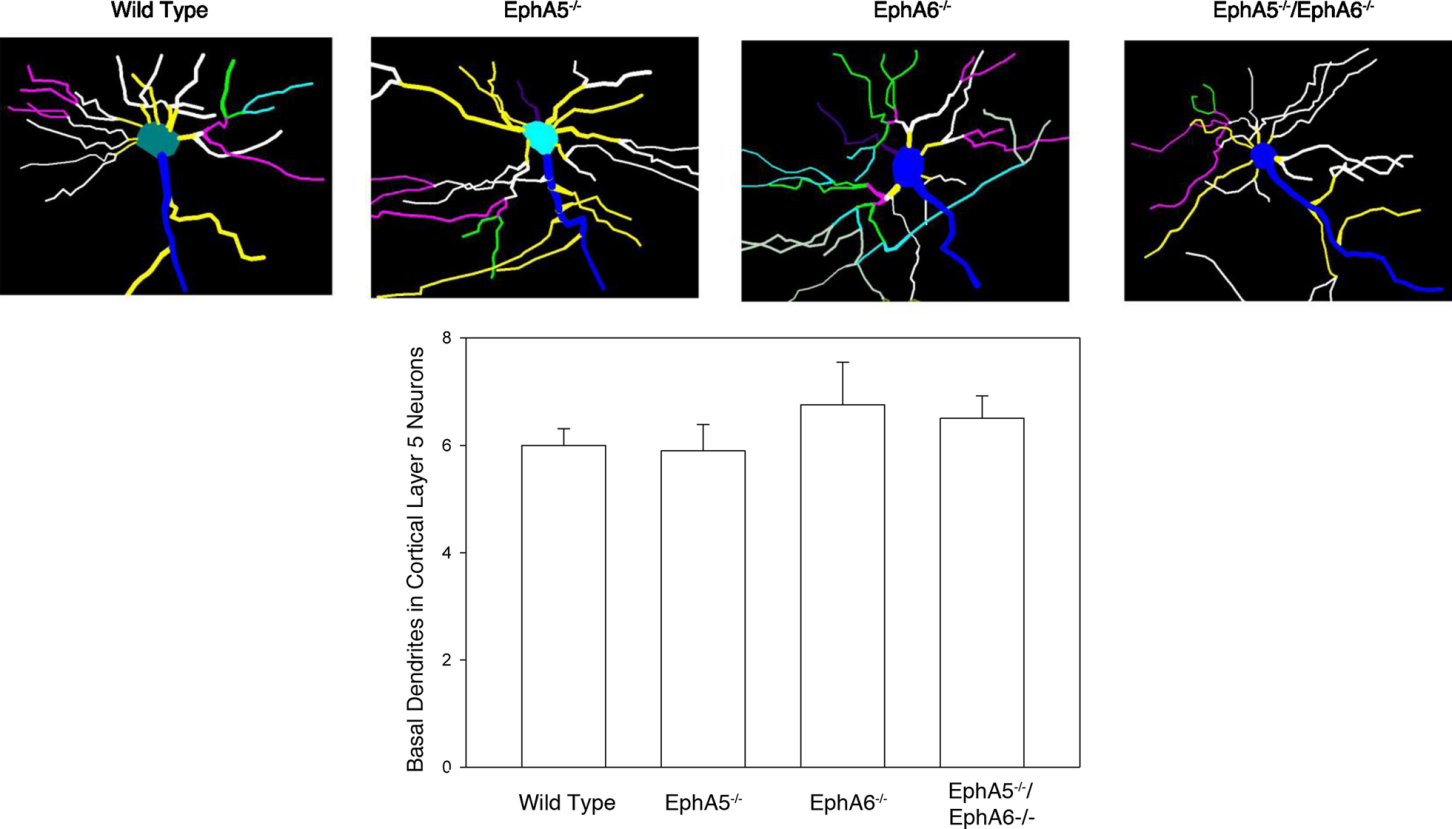
Representative neurolucida drawings of the basal dendrite of the different genotypes in the cortical layer 5 (*upper panel*). Statistical analysis showed no difference in the number of basal dendrites amongst different genotypes (*lower panel*). Total of 60 neurons of each genotype was used for the analysis (three animals, 20 neurons each)

As strong clumping phenotype was observed in cortical layer 5 in all three knockout mouse strains, we wanted to study whether there is also a difference in the average number of the basal dendrites per neuron. We did not observe any significant difference among the genotypes in the number of primary basal dendrites of cortical layer 5 neurons (Fig. 7).

### Spine morphology on the basal dendrite of cortical layer 5 neurons of the EphA5^−/^, EphA6^−/−^ and EphA5^−/−^EphA6^−/−^

**Fig. 8:**
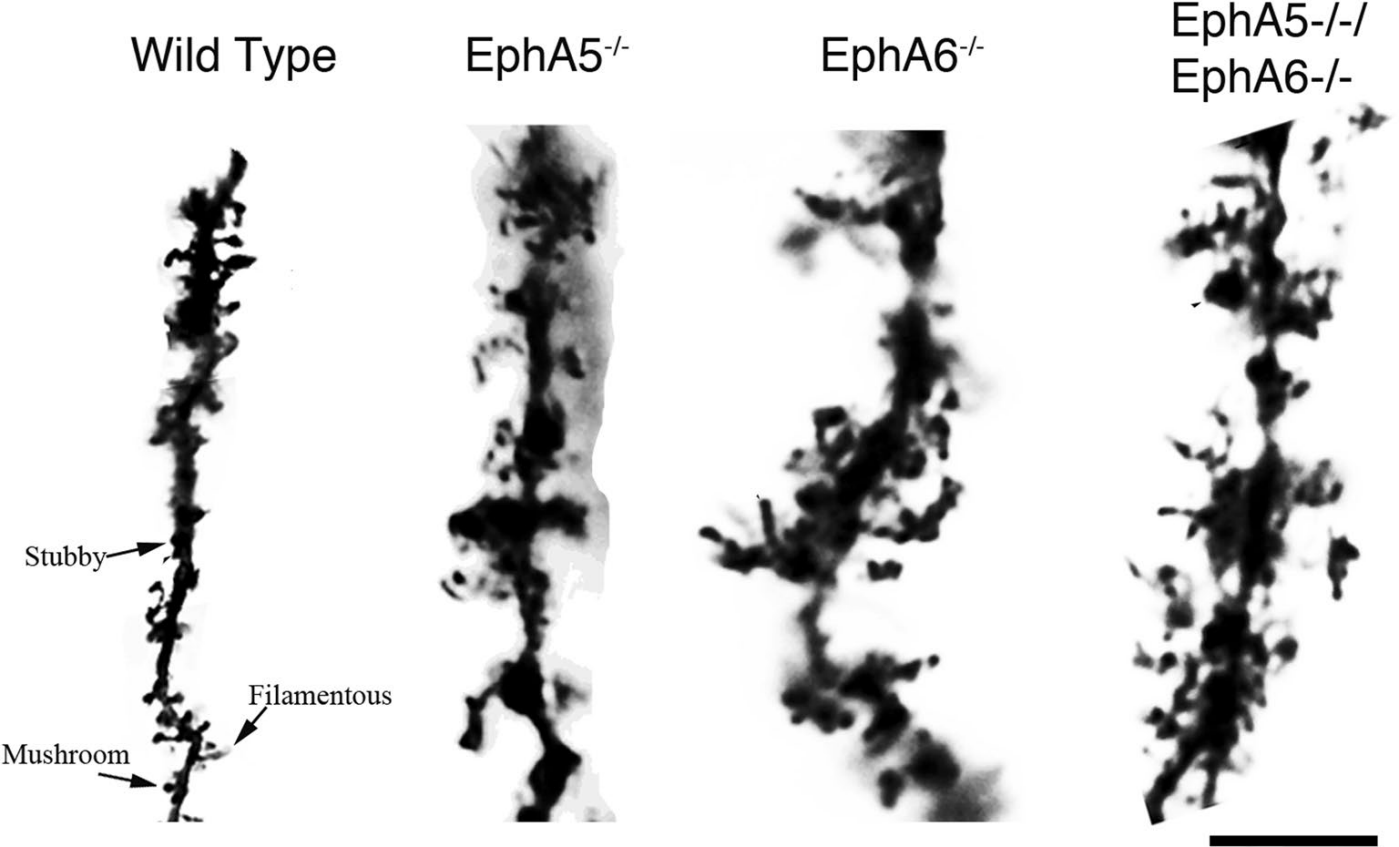
Representative images of spines in the basal dendrites of neurons in cortical layer 5. Wild type spines showed the presence of stubby, filamentous as well as mushroom type of spines, while the knockouts, displaying an irregular morphology, are difficult to classify into the classic categories. *Scale bar* 20 µm

The spines in the basal dendrites of the cortical layer 5 neurons revealed a very interesting and almost bizarre phenotype (Fig. 8) in the knockouts compared to the wild type ones. The wild type spines were very distinct according to their morphological classification as being filamentous, stubby or mushroom like. However, in all the knockouts it was very difficult to classify the spines according to the morphological features. Further, most of the spines in the knockouts formed a flower sort of arrangement with overlaps between different spine types making it very difficult to count the number of spines as well.

## Discussion

We have shown previously that interfering with EphA5 functions by expressing a truncated form of the receptor resulted in deficiencies in spatial learning and active avoidance and a decrease in serotonin concentrations in the striatum [26]. In addition, deletion of EphA5 and one of its ligand, ephrin-A5, caused a defect in attack behavior against intruding male mice [27, 28]. Deletion of EphA6 caused defects in learning and memory impairment. EphA6 KO mice did not freeze as much as wild type mice in a fear conditioning test and had a lower learning score in Morris Water Maze studies [29]. These behavior studies demonstrated a key role of EphA5 and EphA6 receptors in brain function, but the underlying neuronal and pathway deficits have not been elucidated.

This study attempts to determine whether there are specific alternations in the brain in the mice with EphA5 and/or EphA6 deletions. In our Golgi studies, we showed significant morphological abnormalities in the brain of EphA5 and EphA6 knockout animals at 2 months of age, but not at 1 week of age. Thus we examined the expression of EphA6 in the mouse cortex at the age of P0, P10 and 2 months using a knockin genetic marker beta-galactosidase histology method (Fig. 1 and data not shown). We have also examined the detailed expression of EphA5 during development from early embryonic development (E9) to adult previously [25], and re-examined the expression in 2 months-old brains in parallel with EphA6 for comparison (Fig. 1). These analyses showed that significant levels of EphA5 and EphA6 expression were found in the cerebral cortex during perinatal development and in adult mouse brain, suggesting important roles in regulating the cytoarchitecture of the cortical neurons. Ephs are known to act as guidance molecules for migrating neurons and their axons. During development, neurons and their axons sense such cues from the extracellular matrix or nearby cells as guide to travel to their targets and form genetically defined neural maps [33]. The largest group of molecules providing this cue is the Eph/ephrin family of receptor tyrosine kinases. Studies have shown that molecular interaction between Ephs with their corresponding ephrin ligands results in repulsion or attraction of cells and axons depending on the cell types [34–38]. We performed Golgi staining using two different protocols to examine whether EphA5 and EphA6 modulate neuronal as well as non-neuronal cell distribution. Staining of brains prefixed with paraformaldehyde revealed large abnormal cell aggregates in the cerebral cortex of EphA5 and EphA6^−/−^ brains, but not in the similarly treated wild type brains. However, it is known that this procedure also stains glial cells and results in poor detection of dendrites of neurons [31, 39]. We consequently performed the staining with brains without prior fixation. With this procedure, neuronal dendrites are well labeled, and we also observed large cell aggregates of neurons. To further define cell types in the aggregates, we co-immunostained the brain sections with neuron specific marker NeuN with the more general nuclei marker DAPI. This analysis confirms the presence of both NeuN-positive and NeuN-negative cell aggregates, suggesting both neurons and possibly glia cells are present. Thus, in this study, we showed that deletion of EphA5 and/or EphA6 resulted in abnormal cell aggregates in the cerebral cortex, which is not seen in the wild type mouse brain. The double knockouts resembled the EphA6 morphology in general architecture as revealed by Golgi staining suggesting that EphA6 had a more dominant role in regulating cortical cell distribution and patterning.

Although these abnormal cell aggregates are most prominent in parts of the cerebral cortex, they are also found in other brain regions such as the striatum and the hippocampus (Figs. 2, 4). Future studies will be needed to thoroughly map all the areas affected and to determine whether these areas correlate with the receptor expression.

The cellular and molecular mechanisms that underlying these abnormal cell aggregates are currently not clear. These large aggregates may be caused by increased cell aggregation, which would be consistent with previous findings that Eph receptor ligand interaction resulted in cell–cell repulsion [36–38]. Thus, in the absence of EphA5 and EphA6, there is less repulsive activity from their corresponding ephrin ligand-expressing cells, resulting in more adhesive forces among the neurons and glial cells. However, due to the unknown mechanism of Golgi staining, it is also possible that there is increased communication among neurons and glial in the brains of the knockout animals such as increased GAP junctions or other cell junctions. Indeed, previous studies have indicated that Eph signaling inhibited GAP junction functions [40–42]. Future studies are needed to define the exact cellular and molecular mechanisms that result in the presence of these large cell aggregates in the knockout brains.

In this study, we also analyzed the dendritic and spine morphology of basal dendrites in cortical layer 5 neurons as this was the layer of neurons that showed most dramatic effect in both of the knockout strains. We did not observe any significant difference in the number of basal dendrites in the cortical layer 5 neurons. However, the most amazing effect was seen in the morphology of the spines of the layer 5 basal dendrites. In the wild type dendrites, the spines were very well demarcated from each other with clear morphological features that be classified as neck, spine head as also as filamentous, stubby or mushroom. However, in all the knockouts there is no clear distinction between the spine neck and spine head. Moreover, the knockouts showed an abnormal morphology of the spines that cannot be categorized as filamentous, stubby or mushroom. Most spines form large, flowery sort of overlapping structures. Our attempts in quantifying these differences using a number of criteria and shapes including filamentous, stubby or mushroom ran into difficulty because the mutant spine morphology is so drastically incomparable to the normal wild type. Consequently we opted to simply present the images of the Golgi-stained spines to exhibit the changes induced by inactivation of the Eph receptors.

Previous studies have shown that ephrins expressed in the surrounding glial cells help to restrict dendritic growth and promote their maturation in hippocampal neurons [42, 43]. Thus, it is possible that the loss of EphA5/6 results in the loss of inhibitory activity exerted by glia-expressed ephrins, resulting in expanded spines. As spines are the sites of synapse formation, aberration in their morphology and structure will have a significant impact in neuronal functions as demonstrated by previous behavioral studies [26–28, 44]. Further study needs to be done to elucidate the underlying mechanism of such altered spine morphology and their specific effect on associated functions.

## Conclusions

Our findings provide important evidence for the roles of EphA5 and EphA6 in the development of neuronal cytoarchitecture. This study is interesting in that it demonstrates an involvement of EphA5 and EphA6 receptors in both neuronal somata organization and the development of the spine structure.

## Abbreviation

KO: knockout
